# Relating past and present diet to phenotypic and transcriptomic variation in the fruit fly

**DOI:** 10.1186/s12864-017-3968-z

**Published:** 2017-08-22

**Authors:** Christina M. May, Bas J. Zwaan

**Affiliations:** Laboratory of Genetics, Plant Sciences, Wageningen University, Wageningen, 6708 PB the Netherlands

**Keywords:** Predictive adaptive response, Silver spoon hypothesis, Phenotypic plasticity, Longevity, Ribosome, Transcription

## Abstract

**Background:**

Sub-optimal developmental diets often have adverse effects on long-term fitness and health. One hypothesis is that such effects are caused by mismatches between the developmental and adult environment, and may be mediated by persistent changes in gene expression. However, there are few experimental tests of this hypothesis. Here we address this using the fruit fly, *Drosophila melanogaster*. We vary diet during development and adulthood in a fully factorial design and assess the consequences for both adult life history traits and gene expression at middle and old age.

**Results:**

We find no evidence that mismatches between developmental and adult diet are detrimental to either lifespan or fecundity. Rather, developmental and adult diet exert largely independent effects on both lifespan and gene expression, with adult diet having considerably more influence on both traits. Furthermore, we find effects of developmental diet on the transcriptome that persist into middle and old-age. Most of the genes affected show no correlation with the observed phenotypic effects of larval diet on lifespan. However, in each sex we identify a cluster of ribosome, transcription, and translation-related genes whose expression is altered across the lifespan and negatively correlated with lifespan.

**Conclusions:**

As several recent studies have linked decreased expression of ribosomal and transcription related proteins to increased lifespan, these provide promising candidates for mediating the effects of larval diet on lifespan. We place our findings in the context of theories linking developmental conditions to late-life phenotypes and discuss the likelihood that gene expression differences caused by developmental exposure causally relate to adult ageing phenotypes.

**Electronic supplementary material:**

The online version of this article (doi:10.1186/s12864-017-3968-z) contains supplementary material, which is available to authorized users.

## Background

The quality or quantity of available nutrition is a major factor affecting the life history of an organism [1, 2]. The reigning paradigm for studying the effects of nutrition on life histories has been to manipulate diet quality or quantity in a single life stage (e.g. [3–5]). However, in natural settings, organisms are likely to experience environmental variation across multiple life stages [6]. Furthermore, the phenotypic changes that result from environmental conditions in an earlier life stage can potentially influence the range of possible phenotypic responses in later stages (reviewed in [7, 8]). Given the current swift pace of global environmental change, many organisms, including humans, are likely to encounter adult environments markedly different to those in which they developed, highlighting the importance of a more comprehensive understanding of how developmental and adult diets interact [9].

Theories that attempt to link developmental and adult dietary conditions to adult phenotypic variation include the silver spoon [10], the developmental programming [11], and the predictive adaptive response hypotheses [12, 13]. The silver spoon hypothesis proposes that developmental diet affects the overall quality of an individual independent of the adult environment experienced [10]. Thus individuals that develop under poor conditions become poor quality adults with a disadvantage across adult environments, while the opposite is true of individuals that develop under good conditions. For example, water pythons (*Liasis fuscus*) that hatch in seasons with abundant available prey maintain higher growth rates across their lifespan relative to those hatched when prey was scarce [14]. Similar effects have been observed across a broad range of taxa including birds, insects, and mammals [7, 8, 14, 15].

The developmental programming and predictive adaptive response hypotheses propose that the effect of developmental conditions will be dependent on subsequent adult conditions. In the case of developmental programming, individuals make changes to adapt to the current developmental environment which persist into adulthood [11]. If such changes are (largely) irreversible and the adult environment is markedly different from the developmental environment, individuals may be maladapted to their adult environment and have decreased fitness. The PAR hypothesis differs in that the phenotypic changes made during development are not made to adapt to the current environment, but rather in response to cues about the predicted adult environment. The most widely cited example comes from the meadow vole, *Microtus pennsylvanius*. In response to day length, pregnant vole dams emit a hormonal signal that serves as a cue about the season in which the pups will be born. Short day-length cues induce vole pups to develop thicker coats, which will presumably benefit them in winter, while long day-length cues lead to thinner coats [16]. This is considered a PAR because while the differences in coat-length exist at birth, temperatures in utero and in the den remain largely stable year round, and thus the benefit of a thicker (or thinner coat) is only realized once the pup leaves the den [12]. Similar PARs have been proposed to underlie the increased risk of heart disease and other unfavorable metabolic traits in humans born with low birth weight, a proxy for poor nutrition in utero [17–19]. Proposed adaptations include reduced muscle mass, insulin resistance, and a predisposition for storing food as central abdominal fat [20]. It is argued that such changes would be beneficial in a poor quality adult environment, but detrimental in a high-quality one (i.e. a “matched” and “mismatched” environment, respectively).

At a physiological level, developmental conditions can influence adult phenotypes by changing overall size [21], by altering relative investment into different tissues or functions (e.g. [22–24]), and/or by permanently modulating patterns of gene expression. In all cases, these effects are expected to manifest themselves as changes in gene expression at the level of the whole organism. In fact, in the case of predictive adaptive responses (PARs) it is often hypothesized that gene expression changes per se may be the principal cause of long-term phenotypic effects, rather than just being a read-out of past changes [25, 26]. Despite the potential importance of gene expression changes in modulating long-term effects of developmental diet, this area is only beginning to be explored. For example, a restricted-protein maternal diet strongly induces the expression of the transcription factor PPARα at six days of age in rats [27], while in the fruit fly, *Drosophila melanogaster*, high protein larval diets increase the expression of several immune genes in young adults [28]. Furthermore, the expression of hundreds of genes is altered in sexually mature *Drosophila mojavensis* depending on developmental host cactus species and length of development [29]. These studies show that developmental conditions can influence gene expression into adulthood, however, as they focus on gene expression in young or middle-aged adults and in a single adult environment without concomitant measures of fitness-related traits, several open questions remain. These include whether such effects persist into late-life, whether they are dependent on adult conditions as predicted by the PAR and programming hypothesis, and whether they are related to adult phenotypic variation.

**Fig. 1:**
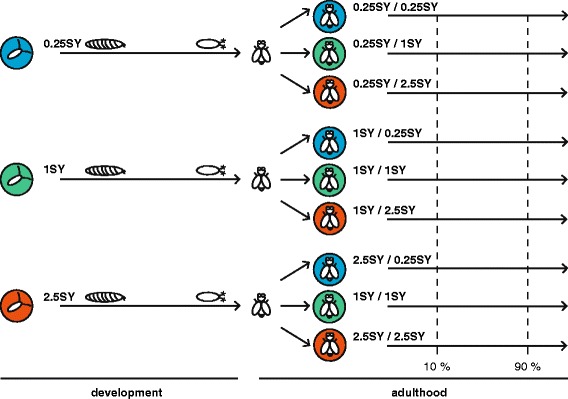
Experimental design. Eggs developed from larvae to adults under three diets, 0.25SY, 1SY and 2.5SY, that differed only in their concentrations of sugar and yeast. Emerging adults were immediately divided across these same three diets resulting in a total of nine different treatment groups. Gene expression was measured on virgin flies sampled when 10% (middle age) and 90% (old age) of the treatment cohort had died

To address these unanswered questions we used the fruit fly, *Drosophila melanogaster,* as a model to understand how mismatches between developmental and adult diet influence adult phenotypes and gene expression. To this end, we varied the caloric content of both developmental and adult diet 10-fold in a three-by-three full factorial design (Fig. 1), and assessed gene expression at middle and old-age. Because there is considerable evidence that phenotypic responses to diet tend to be sex-specific (e.g. [30]), we phenotyped both male and female lifespan, and female fecundity. This approach had three aims: first, to classify the nature of the phenotypic relationship between developmental and adult diet in the fruit fly, which to our knowledge has not yet been assessed; second, to determine whether and how much developmental diet affects gene expression into middle and old-age, and third, to determine whether there was any discernible link between developmentally-induced changes in gene expression and the adult phenotype. We measured gene expression profiles in whole bodies because there was no a priori hypothesis about which tissues and functions would be involved and lifespan is likely determined by more systemic expression variation.

## Results

### Phenotypic variation is driven by adult diet, but consistently modified by larval diet

We first assessed the extent to which developmental and adult diet affected key adult fitness components. We measured mated and virgin lifespan (both sexes) and mated fecundity (females only). To do so we raised larvae on three diets and randomly distributed the emerging adults across these same three diets in a full factorial design (Fig. 1). The diets were obtained by relative dilution of the sugar (S) and yeast (Y) of our standard laboratory diet (1SY: 70 g sugar, 100 g yeast, 20 g agar, 15 mL nipagin solution and 3 mL propionic acid per liter of water) by 0.25 times (0.25SY) and 2.5 times (2.5SY) times respectively, representing a 10-fold change in sugar and yeast concentration. The three diets are referred to throughout as 0.25SY, 1SY and 2.5SY.

**Fig. 2:**
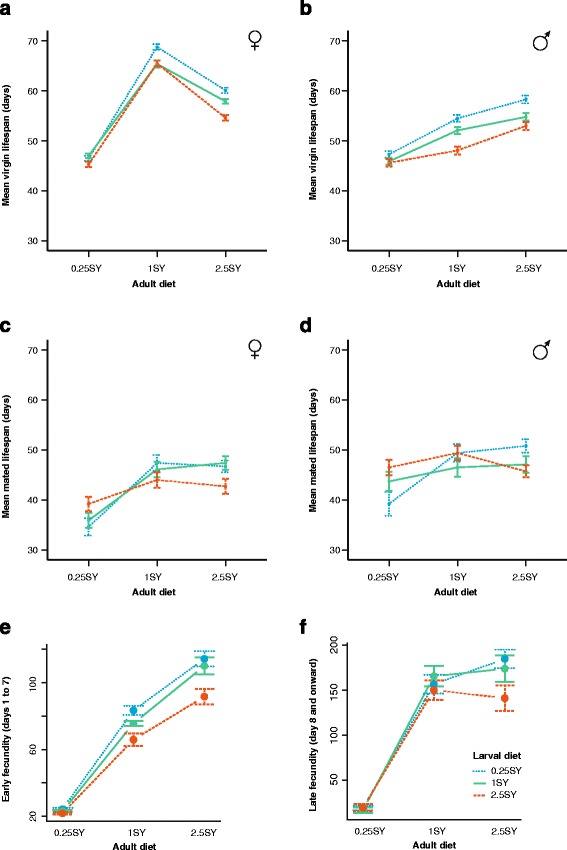
Responses of adult life history traits to adult diet (along x-axis) and larval diet (indicated by colour coding). Mean female (**a**) and male (**b**) virgin lifespan. Mean female (**c**) and male (**d**) mated lifespan. Mean early (**e**) and late (**f**) mated female fecundity. Early fecundity (**e**) encompasses total fecundity from days one to seven of adult life, while late fecundity (**f**) is the total fecundity from day seven to the end of reproduction. All values are means ± standard error

In both sexes, most of the variation in virgin lifespan was attributable to adult diet (Table 1). For females, lifespan peaked on the 1SY adult diet (Fig. 2a), while for males, lifespan increased with increasing adult diet (Fig. 2b). Larval diet, by contrast, explained a smaller, though still highly significant proportion of the variation in virgin lifespan (Table 1). The 0.25SY larval diet tended to increase lifespan across adult diets, while the 2.5SY larval diet decreased it (Fig. 2a,b; *p*-values for all pairwise comparisons between larval diets given in Additional file 1: S1). There was a weak interaction between larval and adult diet in both sexes (Table 1) such that on the 0.25SY adult diet, while the absolute pattern of lifespan differences between larval diets was similar (i.e. 0.25SY > 1SY > 2.5SY) the magnitude of the difference was smaller and did not reach significance for all pairwise contrasts (Additional file 1: S1).

**Table 1:** Analysis of deviance for each phenotype, indicating the relative effect size of adult diet, larval diet and their interaction per sex relative to the null model with no factor effects

Phenotype	Sex	Factor	Log likelihood	χ2	df	*p* value
Virgin Lifespan	Female	Null model	−22,555.16			
		Adult diet (A)	−21,754.99	1600.34	2	<0.001
		Larval diet (L)	−21,679.02	151.94	2	<0.001
		A * L	−21,667.75	22.53	4	<0.001
	Male	Null model	−19,930.17			
		Adult diet (A)	−19,757.87	282.18	2	<0.001
		Larval diet (L)	−19,898.96	62.42	2	<0.001
		A*L	−19,747.86	20.01	4	<0.001
Mated Lifespan	Female	Null model	−4927.58			
		Adult diet (A)	−4894.87	65.42	2	<0.001
		Larval diet (L)	−4893.09	3.55	2	0.171
		A * L	−4886.69	12.79	4	0.012
	Male	Null model	−3919.84			
		Adult diet (A)	−3916.34	3.77	2	0.151
		Larval diet (L)	−3918.23	3.23	2	0.200
		A*L	−3911.46	9.74	4	0.045
Early Fecundity	Female	Adult diet (A)		3100.10	2	<0.001
		Larval diet (L)		94.98	2	<0.001
		A * L		4.49	4	0.343
Late Fecundity	Female	Adult diet (A)		8461.28	2	<0.001
		Larval diet (L)		91.94	2	<0.001
		A * L		53.53	4	<0.001

For Cox proportional hazard models and GLMs with Poisson distribution the chi-squared test is most appropriate

Mated fecundity also depended primarily on the adult diet (Table 1), both early (Fig. 2e) and late in life (Fig. 2f). In the first week of life, adult diet and larval diet both affected fecundity independently but with opposing effects: increasing adult diet increased fecundity across all larval diets (all *p* values <0.0001), while increasing larval diet decreased fecundity across all adult diets (all *p* values <0.01,Fig. 2e). After the first week of adult life, however, the differences in fecundity between larval diets disappeared on the 0.25SY and 1SY adult diets (all *p*-values >0.05), but persisted on the 2.5SY adult diet (all *p*-values <0.02; Fig. 2f), resulting in a significant interaction between larval and adult diet in determining late fecundity (Table 1).

Mated lifespan was much less sensitive to larval and adult diet and showed considerably more variability than virgin lifespan, though it is noteworthy that the cohort size was smaller for the mated flies (100 flies per combination of sex and larval diet for mated lifespan versus 900 flies for mated lifespan; Table 1). In males, adult diet had no overall effect on lifespan, while in females the 0.25SY adult diet shortened lifespan (Females: Fig. 2c; Males: Fig. 2d). In addition, larval diet affected mated lifespan in each sex only under particular adult diets (Additional file 1: S1). Flies raised on the 2.5SY larval diet and subsequently transferred to the 2.5SY adult diet (2.5SY / 2.5SY) had decreased mated lifespan relative to 0.25SY / 2.5SY and 1SY / 2.5SY flies in both sexes (Females: Fig. 2c; Males: Fig. 2d; Additional file 1: S1). In females they also had decreased lifespan under 1SY adult conditions (Fig. 2c; Additional file 1: S1).

Overall, there was no evidence that fitness (as approximated by lifespan and/or fecundity) was negatively affected by mismatches between developmental and adult environments, opposing both the PAR and programming hypotheses. While there were significant interactions between larval and adult diet for all traits except early fecundity, these interactions were of magnitude and not of sign: for example, for virgin longevity, the interaction between larval and adult diet is caused by similar, but smaller differences between larval diet on the 0.25SY adult diet, than on the other two adult diets. The same is true of the other phenotypes and interactive effects: when larval diet has a significant effect, 0.25SY-raised flies perform better than 2.5SY flies, with 1SY-raised flies intermediate between the two. This suggests that the low calorie (0.25SY) diet is beneficial under the range of conditions we tested, while high calorie (2.5SY diet) is detrimental. This tendency for the effects of developmental diet to be consistent across adult diets is therefore consistent with the silver spoon hypothesis [10].

### Pervasive transcriptomic signatures of adult diet and age, and subtle effects of larval diet

We next assessed the relative contributions of larval and adult diet to transcriptional variation in middle and old-aged flies. From the same cohort used to measure virgin lifespan, we sampled flies when 10% (middle-age) and 90% (old-age) of the cohort had died (Fig. 1). We collected four biological replicates of five flies each per combination of sex, larval diet, adult diet, and age (3 larval diets * 3 adult diets * 2 ages * 2 sexes * 4 replicates = 144 samples in total) and hybridised them to Affymetrix Drosophila 2.0 GeneChip microarrays (see Materials and Methods).

To identify the most important contributors to global transcriptional variation we used principal component analysis (PCA; [31]) and principal variance components analysis (PVCA; [32]). PVCA is a supervised version of PCA that estimates the proportion of global transcriptional variation explained by treatment factors. To identify the individual transcripts affected by larval diet, adult diet, and age we fitted an ANOVA model following Ayroles et al., [33]. For each transcript the model partitioned the variation in expression between larval diet (L), adult diet (A), and age (T), as well as the interactions between these factors (L ^x^ A; L ^x^ T; A ^x^ T; L ^x^ T ^x^ A). We then filtered the data to obtain lists of genes affected by each of these factors at a false discovery rate of 0.05 (FDR; [34]).

**Fig. 3:**
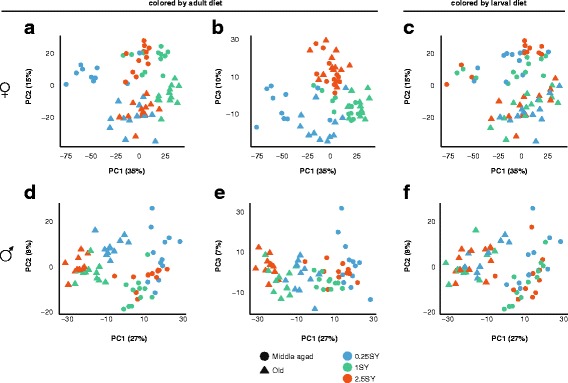
Global patterns of gene expression variation across the lifespan in response to larval diet, adult diet, and age. Scatterplots of PC1 versus PC2 (**a**, **c**, **d**, **f**) and PC1 versus PC3 (**b**, **e**) in females (*top row*) and males (*bottom row*). In (**a**, **b**, **d** and **e**) samples are coloured by adult diet and shape represents age, while in (**c**) and (**f**) the colour indicates larval diet. In females, PC1 roughly separates samples by adult diet (**a**), which is especially visible when plotted against PC3 (**b**). PC2 separates female samples by age (**a**). In males PC1 separates samples by age and within each age class also roughly separates samples by adult diet (**d**). PC2 in males further segregates flies living on the 0.25SY adult diet from the rest (**d**). When colouring PC1 versus PC3 by larval diet (**c** and **f**), no clear grouping is visible

Plotting PC1 against PC2 (Females: Fig. 3a; Males Fig. 3d) and PC1 versus PC3 (Females: Fig. 3b; Males Fig. 3e), samples could clearly be grouped by age (middle and old-age) and adult diet, but not by larval diet (Females Fig. 3c; Males Fig. 3f). In females, PC1 divided flies living on the 0.25SY adult diet from those on 1SY, while flies living on the 2.5SY adult diet fell in between and did not form a distinct cluster (Fig. 3a). PC2 separated middle-aged females (circles) from old females (Fig. 3a), and PC3 separated females living on the 2.5SY adult diet from those on 0.25SY and 1SY (Fig. 3b). In males, the largest component of variation (PC1) separated young males (circles) from old males (triangles) and within these age-classes also separated males roughly by their adult diet (Fig. 3d). PC2 further separated males living on the 0.25SY adult diet from the rest (Fig. 3d). No clear pattern emerged when plotting PC1 against PC2 and coloring samples by larval diet (females: Fig. 3c; males: Fig. 3f), suggesting that while age and adult diet have relatively global effects on the transcriptome, the same is not true of larval diet.

The ANOVA and PVCA analyses broadly confirmed the patterns observed in the PCA (Table 2). In both sexes, most of the variation in expression overall (PVCA) and at a per-transcript level (ANOVA) was due to adult diet and age. PVCA attributed 32.5 and 22.7% of the total observed variation in females to adult diet and age respectively, and 15.5 and 35.6% in males. ANOVA identified 4504 and 3589 transcripts affected by adult diet and age in females and 2688 and 6111 transcripts in males (Table 2). Thus the relative importance of adult diet and age is reversed between the sexes, the transcriptome being more strongly affected by adult diet in females and age in males (Table 2). However, in females there were considerably more transcripts showing an interaction between adult diet and age (TA; 3832 transcripts) than in males (TA; 806 transcripts).

**Table 2:** Overall effects of larval diet, adult diet, age, and their interaction on global patterns of transcriptional variation as determined by principal variance components analysis (PVCA) and ANOVA analysis

	Male			Female		
Factor	PCAV (%)	Probes	% of probes	PCAV (%)	Probes	% of probes
Larval diet(L)	4.4	1999	19.7	1.1	321	2.7
Adult diet(A)	15.5	2688	26.6	32.5	4504	38.5
Age(T)	35.6	6111	60.4	22.7	3589	30.7
LA	1.1	140	1.4	1.5	770	6.6
LT	0.8	240	2.4	0.7	170	1.5
TA	4.1	806	8.0	8	3832	32.8
LTA	-	288	2.8	-	464	4.0
Residual	38.6	-	-	33.5	-	-

The “% of probes” category shows the percentage of probes affected as a fraction of the total number of probes affected by any factor in each sex and sums to more than 100% because many probes show effects of several factors (Males: 10,164 probes; Females: 11,695)

ANOVA analysis also identified effects of larval diet on the transcriptome, both as a main effect and in interaction with the other treatments. In both sexes these factors affected fewer transcripts than the factors adult diet or age (Table 2). In total, there were 2667 and 1725 transcripts affected by larval diet, either as a main effect or in interaction with other factors, in males and females respectively. In males, the majority of transcripts showed a main effect of larval diet (1999 transcripts), while in females, the largest group of transcripts had expression patterns reflecting an interaction between larval and adult diet (770 transcripts; Table 2).

### Identifying links between longevity and transcriptional variation

After quantifying the relative effect of larval versus adult diet on the transcriptome, our next aim was to address whether we could link the observed effects of larval diet on virgin lifespan to the gene expression changes identified in the ANOVA analysis. We applied K-means clustering to identify groups of transcripts affected by larval diet and showing similar expression profiles. We then compared the expression profiles of these clusters to the observed effects of larval diet on lifespan.

Because the effects of larval diet on virgin lifespan were similar in each sex and across adult diets, the simplest hypothesis is that the genes involved would also have similar expression profiles across adult diets and across ages. Indeed, given the consistent phenotypic responses it is difficult to envision how such main effects could be attributable to interactions between larval diet and adult diet and/or age (though this possibility cannot be definitively excluded). Thus we focus on the transcripts showing a main effect of larval diet (L), and provide the results for the transcripts showing an interaction (LT, LA, LTA) in Additional file 2: S4, Additional file 3: S5, and Additional file 4: S6 respectively. It is noteworthy that most of the interactive effects are due to flies raised on the 0.25SY larval diet showing distinct responses to adult diet, age or both. Thus, while these interactions may potentially explain the increased lifespan of 0.25SY-raised flies, it is unlikely that they account for the lifespan differences observed between 1SY and 2.5SY-raised flies.

### The expression of ribosome-related transcripts is positively correlated with larval diet in females

**Fig. 4:**
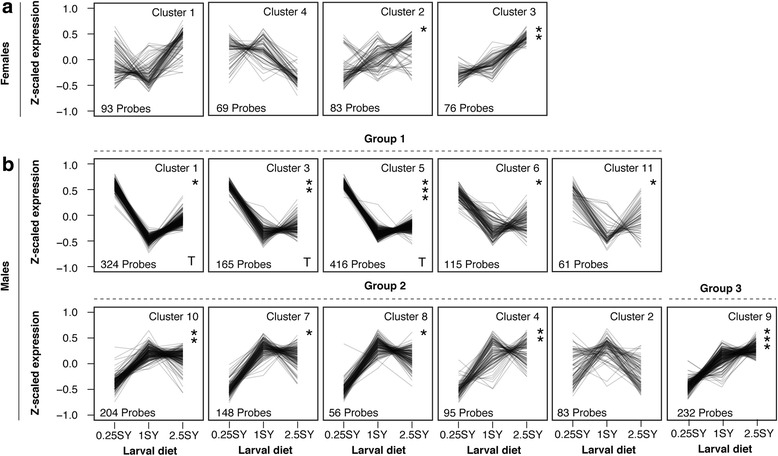
Expression profiles of clusters of probes affected by larval diet across adult diets and age classes in females (**a**) and males (**b**). In females (**a**), Clusters 1 and 4 were not associated with any significant enrichment of gene ontology (GO) terms or tissue-specific genes. Cluster 1 had variable expression in 0.25SY, low expression in 1SY, and high expression in 2.5SY-raised flies, while cluster 4 had roughly the opposite expression pattern. Expression of Cluster 2 and Cluster 3 in particular increases with increasing larval diet. Both clusters were enriched for genes annotated with GO terms relating to ribosomes. In males (**b**), clusters can be roughly classified into three different groups showing similar expression profiles. Clusters in the first group (Group 1) are most highly expressed in 0.25SY-raised flies and least expressed in 1SY-raised flies, while 2.5SY-raised flies fall in between. They differ primarily in the tightness of co-expression, with 2.5SY-raised flies showing the most variation. Clusters 1, 3 and 5 in Group 1 are very significantly enriched for genes with testes-specific expression, and for testes-specific GO terms and contain nearly half of all probes affected by larval diet in males. The second group (Group 2) shows the inverse expression profile of Group 1 and clusters also differ primarily in the tightness of co-expression, especially in 2.5SY-raised flies. The last group (Group 3) consists of one cluster whose expression is positively correlated with increasing larval diet. This cluster significantly overlaps in cluster composition with female cluster 3 (**a**) as well as sharing a similar expression profile and GO enrichment for terms related to ribosomes and transcription and translation. Clusters of probes with similar expression profiles were identified using K-means clustering. * indicates number of GO terms with Benjamin-corrected *p*-value <0.05 associated with a cluster *:1 term, **: 2 to 9 terms,***: 10 or more terms

K-means clustering of the relatively small list of genes showing a main effect of larval diet in females (321 genes, “L” list) identified four clusters, none of which were enriched for genes with tissue-specific expression profiles (Fig. 4a; Additional file 5: S3). Clusters 1 and 4 were not associated with any significant enrichment of gene ontology (GO) terms or tissue-specific genes (Additional file 5: S3 and Additional file 6: S2). Cluster 1 had variable expression in 0.25SY, low expression in 1SY, and high expression in 2.5SY-raised flies, while cluster 4 had roughly the opposite expression pattern (Fig. 4a). The expression of genes in the two remaining clusters, Clusters 2 and 3, increased with increasing larval diet, a pattern particularly evident for cluster 3 (Fig. 4a). Both clusters were enriched for genes annotated with GO terms relating to ribosomes. Cluster 2 (83 transcripts) was solely annotated with the term cytosolic ribosome while cluster 3 (76 transcripts) was annotated with the terms RNA modification, ribonucleoprotein complex, non-coding (nc) RNA metabolic process, non-coding (nc) RNA processing, ribosome biogenesis, ribonucleoprotein complex biogenesis, and cytosolic large ribosomal subunit (Table 3), and contained several sub-units of the 60s large ribosomal sub-unit (RpL3, RpL18, RpL7-like, RpL22 and RpL34a).

**Table 3:** Gene ontology term annotation of male Cluster 9 and female Cluster 3. Italics indicate GO terms that are significant in both clusters. BP: Biological process; CC: Cellular component; MF: Molecular function. Benjamini-corrected *p*-value

Cluster	Category	GO ID	Term	*P*-value
C9 Male	BP	GO:0006396	RNA processing	<0.001
	BP	GO:0034660	ncRNA metabolic process	<0.001
	BP	GO:0034470	ncRNA processing	<0.001
	MF	GO:0000166	nucleotide binding	<0.001
	CC	GO:0031981	nuclear lumen	<0.001
	MF	GO:0001882	nucleoside binding	<0.001
	BP	GO:0016071	mRNA metabolic process	<0.001
	MF	GO:0005524	ATP binding	<0.001
	MF	GO:0032559	adenyl ribonucleotide binding	<0.001
	BP	GO:0006397	mRNA processing	<0.001
	MF	GO:0030554	adenyl nucleotide binding	<0.001
	MF	GO:0001883	purine nucleoside binding	<0.001
	BP	GO:0006399	tRNA metabolic process	<0.001
	CC	GO:0070013	intracellular organelle lumen	<0.001
	CC	GO:0043233	organelle lumen	<0.001
	CC	GO:0031974	membrane-enclosed lumen	<0.001
	MF	GO:0017076	purine nucleotide binding	<0.001
	MF	GO:0032553	ribonucleotide binding	<0.001
	MF	GO:0032555	purine ribonucleotide binding	<0.001
	BP	GO:0022613	ribonucleoprotein complex biogenesis	0.002
	MF	GO:0003723	RNA binding	<0.001
	BP	GO:0008380	RNA splicing	0.004
	BP	GO:0042254	ribosome biogenesis	0.004
	MF	GO:0004386	helicase activity	0.001
	CC	GO:0005730	nucleolus	0.003
	MF	GO:0008186	RNA-dependent ATPase activity	0.003
	MF	GO:0004004	ATP-dependent RNA helicase activity	0.003
	BP	GO:0006364	rRNA processing	0.012
	BP	GO:0016072	rRNA metabolic process	0.012
	BP	GO:0006360	transcription from RNA polymerase I promoter	0.021
	CC	GO:0005654	nucleoplasm	0.011
	BP	GO:0006418	tRNA aminoacylation for protein translation	0.022
	BP	GO:0043039	tRNA aminoacylation	0.022
	MF	GO:0016875	ligase activity, forming carbon-oxygen bonds	0.007
	MF	GO:0016876	ligase activity, forming aminoacyl-tRNA	0.007
	MF	GO:0004812	aminoacyl-tRNA ligase activity	0.007
	BP	GO:0043038	amino acid activation	0.023
	MF	GO:0016779	nucleotidyltransferase activity	0.007
	MF	GO:0034062	RNA polymerase activity	0.006
	MF	GO:0003899	DNA-directed RNA polymerase activity	0.006
	MF	GO:0003724	RNA helicase activity	0.006
	BP	GO:0035196	gene silencing by miRNA, production of miRNAs	0.026
	BP	GO:0035195	gene silencing by miRNA	0.028
	MF	GO:0008026	ATP-dependent helicase activity	0.013
	MF	GO:0070035	purine NTP-dependent helicase activity	0.013
	BP	GO:0000398	nuclear mRNA splicing, via spliceosome	0.047
	BP	GO:0000377	RNA splicing, via transesterification reactions with	0.047
	BP	GO:0000375	RNA splicing, via transesterification reactions	0.047
	MF	GO:0003729	mRNA binding	0.015
	MF	GO:0042624	ATPase activity, uncoupled	0.018
	MF	GO:0004540	ribonuclease activity	0.021
	MF	GO:0032549	ribonucleoside binding	0.029
	MF	GO:0003677	DNA binding	0.031
	MF	GO:0000287	magnesium ion binding	0.038
	MF	GO:0016887	ATPase activity	0.040
C3 Female	BP	GO:0009451	RNA modification	0.002
	CC	GO:0030529	ribonucleoprotein complex	0.001
	BP	GO:0034660	ncRNA metabolic process	0.004
	BP	GO:0034470	ncRNA processing	0.003
	BP	GO:0042254	ribosome biogenesis	0.006
	BP	GO:0022613	ribonucleoprotein complex biogenesis	0.027
	CC	GO:0022625	cytosolic large ribosomal subunit	0.020

### Larval diet affects expression of testes-specific genes in males

In males we identified 11 clusters of genes in the “L” list. These grouped into three different expression profiles (Fig. 4b). The first group (Clusters: 1, 3, 5, 6, and 11) contained more than 50% of the transcripts affected by larval diet (1081 transcripts) and was characterized by high expression in 0.25SY-raised flies, low expression in 1SY-raised flies, and intermediate expression in 2.5SY-raised flies (Fig. 4b). The clusters differed primarily in the tightness of co-expression, especially in 2.5SY-raised flies. The second group (Clusters 10, 7, 8, 4 and 2) was notable for having the inverse expression profile of Group 1 (Fig. 4b). These clusters also differed primarily in the tightness of co-expression, again, especially in 2.5SY-raised flies. The third group consisted of only a single cluster (Cluster 9), which was the only cluster to show a (positive) expression correlation with increasing larval diet (Fig. 4b).

Comparing the male clusters to tissue-specific gene expression data from the FlyAtlas database [35] showed that three of the male clusters (C1, C3, and C5 - all in Group 1) were highly enriched for (1) transcripts exclusively expressed in the testes (hypergeometric test: all *p*-values <0.0001; Additional file 6: S2), (2) transcripts most differentially expressed in the testes relative to the whole body, and (3) transcripts most highly expressed in the testes in the FlyAtlas data set (all *p*-values <0.01). The GO terms associated with these clusters also overlap with those enriched for transcripts highly expressed in the testes in FlyAtlas (C1: 100% overlap, C3: 67% overlap, C5: 96% overlap; Additional file 6: S2). Thus a substantial amount of the long-term effect of larval diet on the transcriptome in males is in genes with testes-specific expression and function, and may explain why we identified considerably less genes affected by larval diet in females (that have no testes). The two remaining clusters in Group 1 (Clusters 6 and 11) show no evidence of tissue-specific expression and are solely annotated with the GO terms neurological system process (C6) and intracellular non-membrane bound organelle (C11; Additional file 1: S1). The second group of clusters (Clusters 10, 7, 8, 4 and 2; Fig. 4b), included most of the remaining transcripts (818 transcripts). None of these clusters showed any significant overlap with genes with testes-specific expression (all *p*-values >0.94), nor with any other tissue. They were enriched for GO terms related to mitochondrial ribosomes (C4), serine-type peptidase activity (C7), transcription (C8), and the break-down of peptidoglycan bonds and immune function (C10; all terms in Additional file 6: S2). The lack of overlap in GO terms among these clusters suggests that they represent an array of different processes.

The third expression profile we identified consisted of one cluster (Cluster 9; Fig. 4b) that contained transcripts whose expression increased with increasing larval diet (Cluster 9; 232 transcripts, Fig. 4b). It was not enriched for tissue-specific transcripts (Additional file 6: S2) and was annotated with 56 GO terms related to ribosome structure, function, and regulation, as well as to other aspects of transcription and translation including tRNA metabolic activity and RNA polymerase activity (Table 3). Furthermore, it contained sub-units of all three eukaryotic RNA polymerases’ (RpII140, RpI135, RpIII128 and RpI1). The probe composition of this cluster overlapped significantly with that of female cluster 3 (Hypergeometric test; *p* < 0.0001), which showed a similar response to larval diet. The overlapping genes were *CDKAL1-like*, *CG32409*, *CG6769*, *Notchless* (nle) and *Elongator complex protein 2* (elp2). All five of these genes are either annotated with GO terms related to ribosome biogenesis and function (ribosome biogenesis: CG32409; ribosomal large subunit biogenesis: CG6769) or are known to play crucial roles in ribosome biogenesis (nle; [36, 37]) or in transcription and translation (elp2; [38, 39]) (CDKAL-1; [40]). Furthermore, of the seven GO terms associated with female Cluster 3, four were also associated with male Cluster 9. These terms were ncRNA metabolic process, ncRNA processing, ribosome biogenesis, and ribonucleoprotein complex biogenesis (Table 3). Taken together, these results suggest that the up-regulation of genes involved in ribosome biogenesis and transcription and translation is an effect of increasing larval diet shared by both sexes.

## Discussion

### *Phenotypic responses to developmental diet in Drosophila melanogaster follow the silver spoon hypothesis*

Our first aim in this study was to determine the nature of the relationship between developmental and adult diet in fruit flies, particularly with respect to the silver spoon [10], programming [41], and predictive adaptive response hypotheses [12]. The former predicting that developmental diet will have a similar effect on adult phenotypes across environments, while the two latter hypotheses predict that the effect will depend on the degree of mismatch between the predicted and actual adult conditions. Our finding that all larval diets show a similar response to adult dietary conditions, particularly in terms of virgin lifespan and early fecundity, is most consistent with the predictions of the silver spoon hypothesis. In fact, we see that flies raised on the different larval diets differ only in their average performance, with 0.25SY-raised flies tending to live longer (Fig. 2a,b) and reproduce more in early life (Fig. 2e), than those raised on the 2.5SY larval diet.

Importantly, adult diet explained considerably more variation than larval diet for both phenotypes (Fig. 2) and gene expression (Fig. 3; Table 2). Thus while larval diet does result in long-term changes in phenotypes and gene expression, extensive plasticity is maintained into adulthood. This suggests that there is no evolutionary advantage to inflexibly “setting” a phenotype to predicted adult conditions in *D. melanogaster*, especially because environmental conditions may be unpredictable throughout the life course of individuals. Rather, it is more likely that the effect of developmental diet on adult phenotypes is due to the persistence of changes that either increased fitness during development, or were unavoidable consequences of a (sub-optimal) developmental environment. Such explanations have also been proposed as alternatives to the PAR hypothesis as applied to humans [42, 43], and are supported by several studies in human cohorts (e.g. [44, 45]). In any case, the absence of PARs in flies does not give any direct information about their plausibility in humans, however, the mechanisms underlying the effect may be similar, even if the adaptive significance of the response differs.

### Long-term effects of larval diet on the transcriptome

Despite the lack of evidence for PARs, we identified long-term effects of larval diet on the transcriptome (Table 2). Given our use of whole flies such effects could be due to any combination of changes in the relative size or function of tissues (e.g. [22–24]), and/or long-term changes in gene expression regulation [46]. Changes in relative tissue size seem especially plausible in the case of the genes we identify with testes-specific expression (Fig. 4b; Clusters C1, C3 and C5). The relative size of the genital arch in *Drosophila melanogaster* is known to decrease as the quality of developmental diet increases [24, 47]. If such effects extend to internal sexual organs such as the testes, this could give rise to the changes in whole-body gene expression we observe. Because we used whole bodies, a relative up-regulation of testes-specific genes would by definition mean a relatively smaller proportion of non-testes mRNA in the whole body pool. This is one potential explanation for the nearly perfect inverse expression profile of the Group 2 clusters relative to Group 1 (which contains C1, C3 and C5; Fig. 4b). These clusters may simply reflect the “down-regulation” of other processes as a result of up-regulation of testes specific genes or relatively larger testes. This is supported by the lack of similarly in GO annotation between the Group 2 clusters, suggesting that they represent a variety of functions (Additional file 6: S2).

The holometabolous nature of *D. melanogaster* suggests another potential mechanism that could translate developmental diet into late-life changes in gene expression: the carry over of larval tissues into adulthood. While it was long thought that all larval tissues were histolysed during development, it is becoming increasingly clear that certain tissues survive pupation and are carried over into adulthood. These tissues include the visceral musculature of the gut [48], the larval fat body [49–51], the malpighian tubules [52], and some neurons [53, 54]. While the larval fat body dissociates and does not persist past the first week of adulthood [50, 51], it is possible that the other tissues or (stem) cells of those tissues are maintained across the lifespan and thus their gene expression and size may depend on developmental conditions. It bears further enquiry to see whether the carry over of larval tissues plays a role in mediating the long-term effects on gene-expression. If so, this provides a potentially more useful parallel to the human case, where tissues are constructed during development and persist throughout the lifespan.

While the effect of larval diet on adult phenotypes was consistent in sign across adult environments and across sexes, most of the transcripts affected by larval diet show no linear relationship with the phenotypes, either because they were involved in an interaction with adult diet, age or both (Additional file 2: S4 to Additional file 4: S6), or because they showed a main effect of larval diet, but not in a direction consistent with the phenotypic effect of diet. For example, ten of the eleven transcript clusters affected by larval diet in males were characterised by intermediate expression in flies raised on the rich larval diet, while in terms of phenotypes, these flies were both most short-lived (males and females) and least fecund (females). This suggests that if the expression of these transcripts is related to lifespan then the relationship between their expression and lifespan is non-linear and complex (e.g. [55–57]).

The ability to infer that the relationship between these 10 transcript clusters and lifespan must be non-linear, if such a relationship exists at all, is a consequence of including three diets in our design. Had we included only two in a classical case-control analysis we would have reached contrasting conclusions on the relationship between gene expression and lifespan for these ten clusters. For example if we used 0.25SY and 1SY-raised flies only, it would have been logical to infer that the genes up and down-regulated in 0.25SY-raised flies relative to 1SY contribute to the phenotypic differences observed between the two, however, had we compared 1SY to 2.5SY, we would have concluded that the relationship between expression levels and phenotypic values was in the opposite direction. The risk of such misguided interpretation in a two-factor design strongly warrants the inclusion of three or more environments, especially when attempting to establish a causal relationship between measures of molecular and/or genetic variation and life history traits (e.g. lifespan, ageing).

We chose to analyze the transcriptomes of virgin, rather than mated females in order to maximize our chances of detecting a clear signature of developmental diet in the adult transcriptome. We rationalized that because the effects of larval diet are more pronounced for virgin, than for mated lifespan (Fig. 2) we would be more likely to detect long-term changes in gene expression. However, understanding whether these results also apply to mated flies is an important area for further study*.* Mating induces differential expression of more than 1700 transcripts in females [58], with more than 500 transcripts affected in the female reproductive tract alone [59]. Since the relative expression of these transcripts changes with time since mating, these changes can induce considerable noise in transcriptome data when samples are taken at time points based on the proportion of the cohort remaining (as in our study). Since mating is a key element of an organism’s life history, it will be important to test whether the gene expression changes we observe for virgin flies can also be detected in mated flies.

### Larval diet affects the expression of transcripts related to ribosomes and translation in both sexes across the lifespan

The consistent effect of larval diet on phenotypes across adult diets and in both sexes suggests that the simplest relationship between gene expression and phenotype is through consistent effects of larval-diet on gene expression across adult diets and in both sexes. Indeed, we identified a single cluster of transcripts showing a linear relationship with larval diet in both sexes (Females C3, Males C9; Fig. 4). Expression of genes in both of these clusters increases with increasing larval diet, across adult diets and age classes, and is thus negatively correlated with the observed lifespan differences (Fig. 4). Furthermore, these clusters have significant overlap in probe composition and shared similar GO annotation, both clusters being enriched for terms relating to ribosomes and ncRNA processing (Table 2), with the male cluster also being enriched for many other terms related to transcription and translation (Table 2). Given the ubiquitous and high expression of ribosomes, accounting for approximately 50% of total transcription [60, 61] and the essential roles of transcription and translation in cellular homeostasis, changes in the regulation of these processes across the lifespan may have important consequences for phenotypes, particularly lifespan. Indeed, in model organisms (primarily flies, yeast, and worms) down-regulation or knock out of ribosomal sub-units results in increased lifespan [62–67], while dysregulation of protein biogenesis machinery is a driver of replicative aging in yeast [68]. Furthermore, down-regulation of translation through the knock-out of various translation initiation factors increases lifespan [64, 69–71].

In eukaryotes, rDNA is typically present in multi-copy arrays, spread across the genome, and only a fraction of the copies in an array are expressed at any given time. Intriguingly, several recent studies, ranging from yeast to mice, have found that diet or nutrient sensing pathways such as TOR can have long-term effects not only on ribosomal expression [61], and methylation [72], but also on copy number [61, 73]. For example, Jack et al., 2015 [73] recently demonstrated that ribosomal copy number in budding yeast is regulated by the TOR pathway, such that increased signaling, indicating nutritional plenty, results in increased rDNA copy number, thereby providing a mechanism linking external nutrient availability to rDNA copy number [73]. In mice, the offspring of dams fed protein-restricted diets throughout gestation and weaning maintain increased rDNA methylation levels into adulthood. This methylation is associated with transcriptional repression and suggests that epigenetic modification of the rDNA array may present yet another way to adjust ribosome expression to nutrient levels, in this case by decreasing mRNA expression in the face of protein restriction [72]. Finally, in *D. melanogaster* increasing levels of dietary yeast during development lead to increased expression of ribosomal RNA genes during development [61] followed by increased rDNA instability and loss of rDNA copies in adults [61]. The loss of rDNA copies is associated with the loss of heterochromatic induced silencing [74] and the loss of heterochromatin in turn de-represses RNA synthesis and decreases lifespan in *Drosophila* [66]. Intriguingly, a very recent study has shown that *D. melanogaster* lifespan can also be programmed by dietary exposure even when the exposure takes place as late as early adulthood. Dobson et al., 2017 [75] showed that a high-sugar diet in early adulthood lead to an irreversible decrease in lifespan, even if diets were quickly changed to lifespan-extending conditions, however, in this case the effect appeared to be mediated by dFOXO.

Taken together, these findings suggest that there is considerable potential for lifespan and gene expression to be set through nutritional conditions experienced early in life, and that this may be mitigated by changes in the expression and modulation of rDNA, potentially as an adaptive way to tune the energetically expensive task of ribosome biogenesis to available energy levels. Our finding that the expression of ribosome and transcription related genes is influenced by larval diet across the lifespan, and that the expression of these genes is negatively correlated with lifespan adds additional support to this theory. Given the simplicity of *Drosophila* as a model system, further research can delve into which nutrient sensing pathways play a role in our particular situation, as both the TOR and insulin signaling pathways have been implicated in the studies discussed above. Furthermore, while we have diluted both sugar and yeast (e.g. protein) simultaneously, additional studies that pinpoint the relative role of each macronutrient in modulating long-term changes in gene expression can further guide research. Such work should be performed within a life history framework as transcription and translation are vital processes that allow flies, and organisms in general, to sustain fitness in their natural environments.

## Conclusions

Here we addressed how developmental and adult diet affect adult phenotypes and gene expression in the fruit fly. We found that for the most part larval and adult diet exert independent effects on the phenotype and on gene expression, and thus there is no evidence for Programming or Predictive Adaptive Responses operating in *Drosophila melanogaster*. Furthermore, adult diet explained considerably more variation in gene expression and phenotypes than larval diet, indicating that flies retain extensive plasticity into adulthood, and this suggests that the long-term effects of developmental diet likely reflect the inability or lack of selection to erase such effects (“a ghost of developmental past”), rather than an adaptive response. We do find that some genes retain a legacy of developmental diet in their expression into middle and old-age. Many of these genes show no correlation with the observed phenotypic responses, however, in both sexes, we identify a cluster of genes whose expression is negatively correlated with the observed lifespan differences and which are enriched with terms related to transcription and translation, particularly with respect to ribosomes. Given several recent studies which show that the down-regulation of ribosomes and other aspects of transcriptional and translational machinery increase lifespan and that rDNA copy number can be influenced by developmental diet, these genes provide promising candidates for mediating the long-term effects of larval diet on lifespan in a laboratory setting. As these processes are highly conserved across the tree of life our results may be relevant for other species as well, including for humans.

## Methods

### Experimental design

To test the effect of mismatches between developmental and adult diet on lifespan, fecundity and gene expression, we raised flies from the laboratory stock population (S), described in May et al., (2015) [70], on three larval diets at a density of 100 eggs per vial. Upon emergence virgin adults were sexed and randomly distributed across these same diets in a three by three full factorial design (Fig. 1). The three diets, hereafter designated 0.25SY, 1SY, and 2.5SY differed only in the amount of sugar and yeast they contained. The 1SY diet was identical to the standard laboratory diet (70 g yeast, 100 g sugar, 20 g agar, 15 mL nipagine, and 3 mL propionic acid per liter of water), while the 0.25SY and 2.5SY diets contained 25% and 250% as much sugar and yeast as the 1SY diet, respectively.

In previous experiments we assessed the effects of these three diets on developmental traits [76]. Flies raised on the 0.25SY larval diet develop more slowly and are smaller as adults than those raised on the 1SY diet, while flies raised on the 2.5SY larval diet are intermediate between the two [76].

### Mated lifespan and fecundity

To assess mated lifespan and fecundity, flies were maintained in groups of five males and five females per vial with 20 replicate vials per treatment. Survival was measured three times per week, while fecundity was scored biweekly over two time periods: early fecundity (days 1 to 7 of adult life), when peak fecundity occurs, and late fecundity (days 7 to end of reproduction) when fecundity is steadily decreasing [77–79]. Fecundity was scored as realized fecundity – i.e. the number of eclosing adults per vial.

### Virgin lifespan and gene expression

To measure virgin lifespan and gene expression, we maintained 500 flies per combination of sex, larval diet and adult diet at a density of ten flies per vial (9000 flies total) and monitored their survival. When 10% and 90% of the cohort had died, 50 flies per combination of sex, larval and adult diet were flash-frozen for gene expression analysis.

We extracted RNA from whole bodies of five flies per replicate, with four replicates per combination of sex, larval diet, adult diet and age (4 replicates × 2 sexes × 3 larval diets × 3 adult diets × 2 ages = 144 arrays), using the Machery Nagel Nucleospin II kit (Machery and Nagel). Biotin labelling, cRNA synthesis, hybridization to Affymetrix *Drosophila* 2.0 GeneChips and array readouts were performed by ServiceXS (www.genomescan.nl).

All statistical analyses were performed using R [80]. Virgin and mated lifespan were analyzed using Cox proportional hazard regression models and fecundity was analyzed using a general linear model (GLM) with a Poisson distribution.

### Gene expression data pre-processing

Prior to normalization we assessed the quality of the arrays and identified outliers using the simpleaffy R package [81] and Principle Component Analysis [31]. Based on these results we excluded two female and four male samples from further analysis due to insufficient quality. These excluded samples were evenly distributed and thus no experimental group contained fewer than three biological replicates. Subsequently we performed background adjustment, quantile normalization and summarization using the robust multi-array average (RMA) algorithm [82] on the remaining 138 samples. We found that when males and female samples were normalized together, 92% of the variation in expression was due to sex, so we chose instead to normalize male and female samples separately to emphasize the effects of diet and age rather than ubiquitous and well-documented sex-specific differences [33]. We performed all subsequent analysis steps separately for each sex using R (version 3.0.0) and Bioconductor [83].

### Assessing the relative contribution of larval diet, adult diet, and age to variation in gene expression

To understand the major factors driving the variation in the transcriptome data we applied principal components analysis [PCA; 31] and principal variance components analysis [PVCA; 32]. PVCA is a supervised version of PCA that partitions the proportion of total variation attributable to treatment factors, and thus estimates the total variation in the gene expression data explained by larval diet, adult diet, age, and their interactions.

To understand the factors driving expression at a per probe level we fitted an ANOVA model to each expressed transcript following Ayroles et al., [33]. For each transcript the model partitioned the variation in expression between larval diet (L), adult diet (A), and age (T), as well as the interactions between these factors (L ^x^ A; L ^x^ T; A ^x^ T; L ^x^ T ^x^ A). We then filtered the data to obtain individual lists of genes affected by each of these factors at a False Discovery Rate of 0.05 [FDR; 34]. For all main effect gene lists (L, A, T) we applied an additional filtering step to remove genes that also showed a significant interaction with another factor. For example, the “L” gene list contains transcripts whose expression was significantly affected by larval diet at an FDR of 0.05, but with no significant interaction between larval diet and any other factors.

### Assessing expression differences between larval diets

We next grouped transcripts affected by larval diet into clusters of genes showing similar expression profiles by applying K-means clustering to the z-score transformed expression values for each sex and for each of the effects (L, L*T, L*A, L*T*A). We then addressed whether the clusters showed any evidence of shared biological function by applying three additional analysis steps to each cluster: first we assessed enrichment of gene ontology (GO) terms using DAVID v6.7 (The Database for Annotation, Visualization and Integrated Discovery; [84]). We focused on “GO FAT” terms, which eliminate term redundancy and increase specificity of gene ontology analysis. Second, we checked for over-representation of transcripts expressed only in particular tissues by using the FlyAtlas database which contains gene expression data from individual *Drosophila* tissues [35]. We filtered the FlyAtlas dataset to extract lists of genes that were exclusively expressed in a single tissue (Additional file 1: S1) and then checked for over-representation of tissue-specific lists in each of the clusters using a hypergeometric test. Finally, we checked for significant overlap in cluster composition between the sexes using a hypergeometric test.

## Additional files

Additional file 1: S1. Pairwise contrasts between larval diets per adult diet for male and female virgin and mated lifespan (Table). (PDF 142 kb)

Additional file 2: S4. Expression profiles of clusters of probes showing an interaction between larval diet and age in females (a) and males (b). Colour indicates larval diet (blue: 0.25SY, green: 1SY, red: 2.5SY) and the x-axis indicates larval diet and age (M-A: middle-aged and O: old). We identified five clusters in females (a) and four clusters in males (b), with no significant overlap between the male and female clusters, and with little significant GO annotation. These clusters revealed that the interaction between larval diet and age in both sexes was primarily due to 0.25SY-raised flies showing attenuated (males) or opposite (females) changes in expression with increasing age relative to 1SY and 2.5SY flies. In males, three of the clusters (C1,C2 & C4) were down-regulated with age in 1SY and 2.5SY-raised flies but up-regulated (C1 – proteasome complex, C4) or unchanging (C2– no annotation) in flies raised on the 0.25SY diet. The remaining cluster C3, was associated with the mitochondrial envelope and was down-regulated with age in 1SY and 2.5SY-raised flies, but relatively constant in 0.25SY-raised flies. In females, all clusters showed relatively little change in expression with age in 1SY and 2.5SY-raised flies, but down (C1,C2, C4) or up-regulation (C3,C5) in 0.25SY-raised flies. The only significant GO annotation was found for C3 (iron ion binding) and C4 (heme binding and terms relating to mitochondrial components and ATP synthesis). M-A: Middle-aged; O: Old age. Clusters of probes with similar expression profiles were identified using k-means clustering. * indicates number of significant GO terms associated with a cluster *:1 term, **: 2: to 9 terms,***: 10 or more terms. (PDF 841 kb)

Additional file 3: S5. Expression profiles of clusters of probes showing an interaction between larval diet and adult diet in males (a) and females (b). Colour indicates larval diet (blue: 0.25SY, green: 1SY, red: 2.5SY). In contrast to the genes affected by larval diet alone (L), there were many more probes showing L*A effects in females (770) than in males (140). In males, these probes broke down into six small clusters, only two of which (Clusters 4 and 5) had significant annotation. Cluster 4, which was up-regulated on the 1SY adult diet in 2.5SY-raised flies was annotated with responses to abiotic stimuli, particularly heat and oxygen, while cluster 5 which was down-regulated on the 1SY adult diet in 2.5SY-raised flies was associated with nucleobase metabolic processes. In females we identified three large clusters, two of which showed very distinct expression patterns and significant annotation. As with the “LT” clusters, these clusters were characterized by very similar expression patterns in 1SY and 2.5SY-raised flies, but distinct patterns in 0.25SY-raised flies. Cluster two was characterized by high expression on the 0.25SY adult diet for 1SY and 2.5SY-raised flies, but low expression in 0.25SY-raised flies. It was strongly associated with visual perception, circadian rhythm, regulation of behaviour, and metal ion transport. Cluster three was characterized by low expression on the 0.25SY adult diet for 1SY and 2.5SY-raised flies, but higher expression in 0.25SY-raised flies. This cluster was associated with nucleotide binding and female reproduction. Clusters of probes with similar expression profiles were identified using k-means clustering. * indicates number of significant GO terms associated with a cluster *:1 term, **: 2: to 9 terms,***: 10 or more terms. (PDF 2348 kb)

Additional file 4: S6. Expression profiles of clusters of probes showing an interaction between larval diet (colour), adult diet (x-axis) and age in males (a) and females (b). Colour indicates larval diet (blue: 0.25SY, green: 1SY, red: 2.5SY), and the panels are split into middle age (M-A) and old-age (O). In males we identified six clusters of which only two possessed significant GO annotation – C4: endoplasmic reticulum and C5: post-mating behaviour. Clusters C1, C4 and C6 are characterized by distinct expression profiles for each larval diet across adult diets at middle-age, which are then inverted in old age, while clusters C3 & C5 are characterized by inversions in expression profiles across adult diets in age classes in 2.5SY-raised flies only. Finally, C2 shows distinct expression profiles in middle-age for 0..25SY & 1SY-raised flies, but no clear expression pattern in other ages or in 2.5SY-raised flies. In females, we identified five clusters each of which displayed distinct expression profiles and only two of which were annotated with GO terms (C3 and C5). As opposed to the males, we saw no evidence of inversion of responses to adult diet with increasing age. Cluster 3, which is associated with peptidase activity shows a distinct expression profile in old-aged flies raised on the 2.5SY larval diet, while cluster 5, which is associated with various terms relating to immune function and development, also shows a distinct expression profile in old-aged flies raised on the 2.5SY larval diet, as well as in middle-aged flies raised on the 0.25SY larval diet. Clusters of probes with similar expression profiles were identified using k-means clustering. * indicates number of significant GO terms associated with a cluster *:1 term, **: 2: to 9 terms,***: 10 or more terms. (PDF 4145 kb)

Additional file 5: S3. Tabbed excel file of hypergeometric tests of overlaps in probe composition between male and female clusters (Tabs 1 to 4) and for overlap in cluster probe composition with tissue-specific probe lists mined from FlyAtlas (Tabs 5 to 12). (XLSX 84 kb)

Additional file 6: S2. Tabbed Excel File of results of ANOVA analysis per probe in females (Tab. 1), Males (Tab. 2). Cluster membership in both sexes (Tab. 3). Tissue-specific gene lists mined from FlyAtlas for females (Tab. 4) and for males (Tab. 5). Significant GO term annotation of all clusters (Tab. 6). Number of overlapping GO terms between tissue-specific gene lists and clusters in females (Tab. 7) and males (Tab. 8). (XLSB 3739 kb)

## Abbreviations

A: Adult diet
GLM: General linear model
GO: Gene ontology
L: Larval diet
PAR: Predictive adaptive response
PC: Principal component
PCA: Principal component analysis
PVCA: Principal variance components analysis
RMA: Robust multi-array average
SY: Sugar-yeast
T: Age

## References

[1] Stearns SC (1992). The evolution of life histories.

[2] Roff DA. Life history evolution. Sunderland: Sinauer associates; 2001.

[3] Tu MP, Tatar M (2003). Juvenile diet restriction and the aging and reproduction of adult drosophila melanogaster. Aging Cell.

[4] Skorupa DA, Dervisefendic A, Zwiener J, Pletcher SD (2008). Dietary composition specifies consumption, obesity, and lifespan in *Drosophila melanogaster*. Aging Cell.

[5] Solon-Biet SM, McMahon AC, Ballard JWO, Ruohonen K, Wu LE, Cogger VC, Warren A, Huang X, Pichaud N, Melvin RG (2014). The ratio of macronutrients, not caloric intake, dictates cardiometabolic health, aging, and longevity in ad libitum-fed mice. Cell Metab.

[6] Schlichting C, Pigliucci M. Phenotypic evolution: a reaction norm perspective. Sunderland: 1998.

[7] Lindström J (1999). Early development and fitness in birds and mammals. Trends Ecol Evol.

[8] Monaghan P (2008). Early growth conditions, phenotypic development and environmental change. Philosophical Transactions of the Royal Society B: Biological Sciences.

[9] Chown SL, Hoffmann AA, Kristensen TN, Angilletta MJ, Stenseth NC, Pertoldi C (2010). Adapting to climate change: a perspective from evolutionary physiology. Clim Res.

[10] Grafen A. On the uses of data on lifetime reproductive success. In: Reproductive success. Edited by Clutton-Brock TH. Chicago: University of Chicago Press; 1988: 454–471.

[11] Fernandez-Twinn DS, Ozanne SE (2006). Mechanisms by which poor early growth programs type-2 diabetes, obesity and the metabolic syndrome. Physiol Behav.

[12] Gluckman PD, Hanson MA (2004). The developmental origins of the metabolic syndrome. Trends in Endocrinology & Metabolism.

[13] Gluckman PD, Hanson MA, Beedle AS (2007). Early life events and their consequences for later disease: a life history and evolutionary perspective. Am J Hum Biol.

[14] Madsen T, Shine R (2000). Silver spoons and snake body sizes: prey availability early in life influences long-term growth rates of free-ranging pythons. J Anim Ecol.

[15] Van de Pol M, Bruinzeel LW, Heg D, Van der Jeugd HP, Verhulst S (2006). A silver spoon for a golden future: long-term effects of natal origin on fitness prospects of oystercatchers (*Haematopus ostralegus*). J Anim Ecol.

[16] Lee TM, Zucker I (1988). Vole infant development is influenced perinatally by maternal photoperiodic history. Am J Physiol Regul Integr Comp Physiol.

[17] Barker D, Winter P, Osmond C, Margetts B, Simmonds S (1989). Weight in infancy and death from ischaemic heart disease. Lancet.

[18] Barker D, Thornburg K (2013). The obstetric origins of health for a lifetime. Clin Obstet Gynecol.

[19] Bateson P, Gluckman P, Hanson M (2014). The biology of developmental plasticity and the predictive adaptive response hypothesis. J Physiol.

[20] Gluckman PD, Hanson MA. The fetal matrix: evolution, development and disease. Cambridge: Cambridge University press; 2005.

[21] Shingleton A (2011). Evolution and the regulation of growth and body size. Mechanisms of life history evolution.

[22] Emlen DJ (1997). Diet alters male horn allometry in the beetle *Onthophagus acuminatus* (Coleoptera: Scarabaeidae). Proc R Soc Lond B Biol Sci.

[23] Jannot JE, Bruneau E, Wissinger SA (2007). Effects of larval energetic resources on life history and adult allocation patterns in a caddisfly (Trichoptera: Phryganeidae). Ecological Entomology.

[24] Shingleton AW, Estep CM, Driscoll MV, Dworkin I (2009). Many ways to be small: different environmental regulators of size generate distinct scaling relationships in *Drosophila melanogaster*. Proc R Soc B Biol Sci.

[25] Burdge GC, Hanson MA, Slater-Jefferies JL, Lillycrop KA (2007). Epigenetic regulation of transcription: a mechanism for inducing variations in phenotype (fetal programming) by differences in nutrition during early life?. Br J Nutr.

[26] Burdge GC, Lillycrop KA (2010). Nutrition, epigenetics, and developmental plasticity: implications for understanding human disease. Annu Rev Nutr.

[27] Lillycrop KA, Phillips ES, Jackson AA, Hanson MA, Burdge GC (2005). Dietary protein restriction of pregnant rats induces and folic acid supplementation prevents epigenetic modification of hepatic gene expression in the offspring. J Nutr.

[28] Fellous S, Lazzaro BP (2010). Larval food quality affects adult (but not larval) immune gene expression independent of effects on general condition. Mol Ecol.

[29] Etges WJ, de Oliveira C, Rajpurohit S, Gibbs AG (2016). Preadult life history variation determines adult transcriptome expression. Mol Ecol.

[30] Magwere T, Chapman T, Partridge L (2004). Sex differences in the effect of dietary restriction on life span and mortality rates in female and male *Drosophila melanogaster*. J Gerontol Ser A Biol Med Sci.

[31] Pearson K (1901). LIII. On lines and planes of closest fit to systems of points in space. The London, Edinburgh, and Dublin Philosophical Magazine and Journal of Science.

[32] Bushel P: pvca: Principal variance component analysis (PVCA). In*.*, vol. R Package Version 1.6.0, R package version 1.6.0 edn; 2013.

[33] Ayroles JF, Carbone MA, Stone EA, Jordan KW, Lyman RF, Magwire MM, Rollmann SM, Duncan LH, Lawrence F, Anholt RRH (2009). Systems genetics of complex traits in *Drosophila melanogaster*. Nat Genet.

[34] Benjamini Y, Hochberg Y (1995). Controlling the false discovery rate: a practical and powerful approach to multiple testing. J R Stat Soc Ser B Methodol.

[35] Chintapalli VR, Wang J, Dow JAT (2007). Using FlyAtlas to identify better *Drosophila melanogaster* models of human disease. Nat Genet.

[36] Ulbrich C, Diepholz M, Baßler J, Kressler D, Pertschy B, Galani K, Böttcher B, Hurt E (2009). Mechanochemical removal of ribosome biogenesis factors from nascent 60S ribosomal subunits. Cell.

[37] Le Bouteiller M, Souilhol C, Beck-Cormier S, Stedman A, Burlen-Defranoux O, Vandormael-Pournin S, Bernex F, Cumano A, Cohen-Tannoudji M (2013). Notchless-dependent ribosome synthesis is required for the maintenance of adult hematopoietic stem cells. J Exp Med.

[38] Otero G, Fellows J, Li Y, de Bizemont T, Dirac AMG, Gustafsson CM, Erdjument-Bromage H, Tempst P, Svejstrup JQ (1999). Elongator, a multisubunit component of a novel RNA polymerase II holoenzyme for transcriptional elongation. Mol Cell.

[39] Glatt S, Müller CW (2013). Structural insights into elongator function. Curr Opin Struct Biol.

[40] Wei F-Y, Suzuki T, Watanabe S, Kimura S, Kaitsuka T, Fujimura A, Matsui H, Atta M, Michiue H, Fontecave M (2011). Deficit of tRNA(Lys) modification by cdkal1 causes the development of type 2 diabetes in mice. J Clin Invest.

[41] Ozanne SE, Hales CN (2004). Lifespan: catch-up growth and obesity in male mice. Nature.

[42] Rickard IJ, Lummaa V (2007). The predictive adaptive response and metabolic syndrome: challenges for the hypothesis. Trends in Endocrinology & Metabolism.

[43] Wells JCK (2012). A critical appraisal of the predictive adaptive response hypothesis. Int J Epidemiol.

[44] Hayward AD, Lummaa V (2013). Testing the evolutionary basis of the predictive adaptive response hypothesis in a preindustrial human population. Evolution, Medicine, and Public Health.

[45] Hayward AD, Rickard IJ, Lummaa V (2013). Influence of early-life nutrition on mortality and reproductive success during a subsequent famine in a preindustrial population. Proc Natl Acad Sci.

[46] Harrison PW, Wright AE, Zimmer F, Dean R, Montgomery SH, Pointer MA, Mank JE (2015). Sexual selection drives evolution and rapid turnover of male gene expression. Proc Natl Acad Sci.

[47] Tang HY, Smith-Caldas MSB, Driscoll MV, Salhadar S, Shingleton AW (2011). FOXO regulates organ-specific phenotypic plasticity in drosophila. PLoS Genet.

[48] Klapper R (2000). The longitudinal visceral musculature of *Drosophila melanogaster* persists through metamorphosis. Mech Dev.

[49] Nelliot A, Bond N, Hoshizaki DK (2006). Fat-body remodeling in Drosophila melanogaster. Genesis.

[50] Aguila JR, Suszko J, Gibbs AG, Hoshizaki DK (2007). The role of larval fat cells in adult *Drosophila melanogaster*. J Exp Biol.

[51] Aguila JR, Hoshizaki DK, Gibbs AG (2013). Contribution of larval nutrition to adult reproduction in *Drosophila melanogaster*. J Exp Biol.

[52] Riddiford LM (1993). Hormones and drosophila development. The development of Drosophila melanogaster.

[53] Lee T, Lee A, Luo L (1999). Development of the drosophila mushroom bodies: sequential generation of three distinct types of neurons from a neuroblast. Development.

[54] Marin EC, Watts RJ, Tanaka NK, Ito K, Luo L (2005). Developmentally programmed remodeling of the drosophila olfactory circuit. Development.

[55] Qu Y, Xu S (2006). Quantitative trait associated microarray gene expression data analysis. Mol Biol Evol.

[56] Lebedeva G, Yamaguchi A, Langdon SP, Macleod K, Harrison DJ (2012). A model of estrogen-related gene expression reveals non-linear effects in transcriptional response to tamoxifen. BMC Syst Biol.

[57] Meyer E, Aspinwall MJ, Lowry DB, Palacio-Mejía JD, Logan TL, Fay PA, Juenger TE (2014). Integrating transcriptional, metabolomic, and physiological responses to drought stress and recovery in switchgrass (***Panicum virgatum****L.*). BMC Genomics.

[58] McGraw LA, Clark AG, Wolfner MF (2008). Post-mating gene expression profiles of female *Drosophila melanogaster* in response to time and to four male accessory gland proteins. Genetics.

[59] Mack PD, Kapelnikov A, Heifetz Y, Bender M (2006). Mating-responsive genes in reproductive tissues of female *Drosophila melanogaster*. Proc Natl Acad Sci U S A.

[60] Warner JR (1999). The economics of ribosome biosynthesis in yeast. Trends Biochem Sci.

[61] Aldrich JC, Maggert KA (2015). Transgenerational inheritance of diet-induced genome rearrangements in drosophila. PLoS Genet.

[62] Kaeberlein M, Powers RW, Steffen KK, Westman EA, Hu D, Dang N, Kerr EO, Kirkland KT, Fields S, Kennedy BK (2005). Regulation of yeast replicative life span by TOR and sch9 in response to nutrients. Science.

[63] Chiocchetti A, Zhou J, Zhu H, Karl T, Haubenreisser O, Rinnerthaler M, Heeren G, Oender K, Bauer J, Hintner H (2007). Ribosomal proteins rpl10 and rps6 are potent regulators of yeast replicative life span. Exp Gerontol.

[64] Curran SP, Ruvkun G (2007). Lifespan regulation by evolutionarily conserved genes essential for viability. PLoS Genet.

[65] Steffen KK, MacKay VL, Kerr EO, Tsuchiya M, Hu D, Fox LA, Dang N, Johnston ED, Oakes JA, Tchao BN (2008). Yeast life span extension by depletion of 60S ribosomal subunits is mediated by gcn4. Cell.

[66] Larson K, Yan S-J, Tsurumi A, Liu J, Zhou J, Gaur K, Guo D, Eickbush TH, Li WX (2012). Heterochromatin formation promotes longevity and represses ribosomal RNA synthesis. PLoS Genet.

[67] McCormick Mark A, Delaney Joe R, Tsuchiya M, Tsuchiyama S, Shemorry A, Sim S, Chou Annie C-Z, Ahmed U, Carr D, Murakami Christopher J (2015). A comprehensive analysis of replicative lifespan in 4,698 single-gene deletion strains uncovers conserved mechanisms of aging. Cell Metab.

[68] Janssens GE, Meinema AC, González J, Wolters JC, Schmidt A, Guryev V, Bischoff R, Wit EC, Veenhoff LM, Heinemann M (2015). Protein biogenesis machinery is a driver of replicative aging in yeast. eLife.

[69] Chen D, Pan KZ, Palter JE, Kapahi P (2007). Longevity determined by developmental arrest genes in *Caenorhabditis elegans*. Aging Cell.

[70] Pan KZ, Palter JE, Rogers AN, Olsen A, Chen D, Lithgow GJ, Kapahi P (2007). Inhibition of mRNA translation extends lifespan in *Caenorhabditis elegans*. Aging Cell.

[71] Hansen M, Taubert S, Crawford D, Libina N, Lee S-J, Kenyon C (2007). Lifespan extension by conditions that inhibit translation in *Caenorhabditis elegans*. Aging Cell.

[72] Holland ML, Lowe R, Caton PW, Gemma C, Carbajosa G, Danson AF, Carpenter AAM, Loche E, Ozanne SE, Rakyan VK (2016). Early-life nutrition modulates the epigenetic state of specific rDNA genetic variants in mice. Science.

[73] Jack CV, Cruz C, Hull RM, Keller MA, Ralser M, Houseley J (2015). Regulation of ribosomal DNA amplification by the TOR pathway. Proc Natl Acad Sci.

[74] Paredes S, Maggert KA (2009). Ribosomal DNA contributes to global chromatin regulation. Proc Natl Acad Sci.

[75] Dobson AJ, Ezcurra M, Flanagan CE, Summerfield AC, Piper MDW, Gems D, Alic N (2017). Nutritional programming of lifespan by FOXO inhibition on sugar-rich diets. Cell Reports.

[76] May CM, Doroszuk A, Zwaan BJ (2015). The effect of developmental nutrition on life span and fecundity depends on the adult reproductive environment in *Drosophila melanogaster*. Ecology and Evolution.

[77] Robertson FW, Sang JH (1944). The ecological determinants of population growth in a drosophila culture. I. Fecundity of adult flies. Proc R Soc Lond B Biol Sci.

[78] Sgro MC, Partridge L, editor: Joseph T: Evolutionary responses of the life history of wild & caught Drosophila melanogaster to two standard methods of laboratory culture. Am Nat 2000, 156(4):341–353.

[79] Novoseltsev VN, Arking R, Carey JR, Novoseltseva JA, Yashin AI (2005). Individual fecundity and senescence in drosophila and medfly. J Gerontol A Biol Sci Med Sci.

[80] Team RC (2013). R: a language and environment for statistical computing.

[81] Wilson C, Miller C (2005). Simpleaffy: a BioConductor package for affymetrix quality control and data analysis. Bioinformatics.

[82] Irizarry RA, Hobbs B, Collin F, Beazer-Barclay YD, Antonellis KJ, Scherf U, Speed TP (2003). Exploration, normalization, and summaries of high density oligonucleotide array probe level data. Biostatistics.

[83] Gentleman R, Carey V, Bates D, Bolstad B, Dettling M, Dudoit S, Ellis B, Gautier L, Ge Y, Gentry J (2004). Bioconductor: open software development for computational biology and bioinformatics. Genome Biol.

[84] Huang DW, Sherman BT, Lempicki RA (2008). Systematic and integrative analysis of large gene lists using DAVID bioinformatics resources. Nat Protocols.

