# Supplementary material for: Relating past and present diet to phenotypic and transcriptomic variation in the fruit fly
Source: BMC Genomics. 2017 Aug 22;18:640. doi: 10.1186/s12864-017-3968-z (PMC5568309; doi:10.1186/s12864-017-3968-z)
Supplement: Supplementary file 1 — Pairwise contrasts between larval diets per adult diet for male and female virgin and mated lifespan (Table). (PDF 142 kb) [file 12864_2017_3968_MOESM1_ESM.pdf]

Phenotype	Adult diet	Larval diet contrast	Hazard Ratio [95% CI]	p value
Male Virgin Lifespan	0.25SY	0.25SY : 1SY	0.80 [0.68,0.94]	0.0056
		2.5SY : 1SY	0.88 [0.75,1.03]	0.12
		2.5SY : 0.25SY	1.10 [0.94,1.29]	0.24
	1SY	0.25SY : 1SY	0.71 [0.60,0.85]	0.0001
		2.5SY : 1SY	1.33 [1.12,1.58]	0.0013
		2.5SY : 0.25SY	1.87 [1.56,2.24]	<0.0001
	2.5SY	0.25SY : 1SY	0.69 [0.59,0.80]	<0.0001
		2.5SY : 1SY	1.11 [0.95,1.29]	0.198
		2.5SY : 0.25SY	1.61 [1.37,1.89]	<0.0001
Female Virgin Lifespan	0.25SY	0.25SY : 1SY	1.00 [0.86,1.16]	0.99
		2.5SY : 1SY	1.37 [1.18,1.60]	<0.0001
		2.5SY : 0.25SY	1.37 [1.17,1.60]	<0.0001
	1SY	0.25SY : 1SY	0.67 [0.57,0.78]	<0.0001
		2.5SY : 1SY	1.33 [1.15,1.55]	0.0002
		2.5SY : 0.25SY	1.99 [1.70,2.34]	<0.0001
	2.5SY	0.25SY : 1SY	0.70 [0.60,0.81]	<0.0001
		2.5SY : 1SY	1.43 [1.23,1.67]	<0.0001
		2.5SY : 0.25SY	2.05 [1.75,2.40]	<0.0001
Male Mated Lifespan	0.25SY	0.25SY : 1SY	1.07 [0.79,1.45]	0.65
		2.5SY : 1SY	0.93 [0.69,1.25]	0.62
		2.5SY : 0.25SY	0.86 [0.63,1.18]	0.36
	1SY	0.25SY : 1SY	0.73 [0.52,1.02]	0.062
		2.5SY : 1SY	0.82 [0.59,1.15]	0.24
		2.5SY : 0.25SY	1.13 [0.82,1.56]	0.46
	2.5SY	0.25SY : 1SY	0.86 [0.62,1.19]	0.37
		2.5SY : 1SY	1.49 [1.09,2.02]	0.011
		2.5SY : 0.25SY	1.73 [1.26,2.37]	0.0007
Female Mated Lifespan	0.25SY	0.25SY : 1SY	0.88 [0.66,1.16]	0.36
		2.5SY : 1SY	0.78 [0.59,1/03]	0.084
		2.5SY : 0.25SY	0.89 [0.68,1.18]	0.42
	1SY	0.25SY : 1SY	0.95 [0.72,1.25]	0.71
		2.5SY : 1SY	1.42 [1.07,1.89]	0.016
		2.5SY : 0.25SY	1.50 [1.11,2.01]	0.0076
	2.5SY	0.25SY : 1SY	1.10 [0.82,1.48]	0.52
		2.5SY : 1SY	1.50 [1.11,2.04]	0.0089
		2.5SY : 0.25SY	1.37 [1.02,1.834]	0.039
