# Supplementary figures and images for: Relating past and present diet to phenotypic and transcriptomic variation in the fruit fly
Source: BMC Genomics. 2017 Aug 22;18:640. doi: 10.1186/s12864-017-3968-z (PMC5568309; doi:10.1186/s12864-017-3968-z)

a.

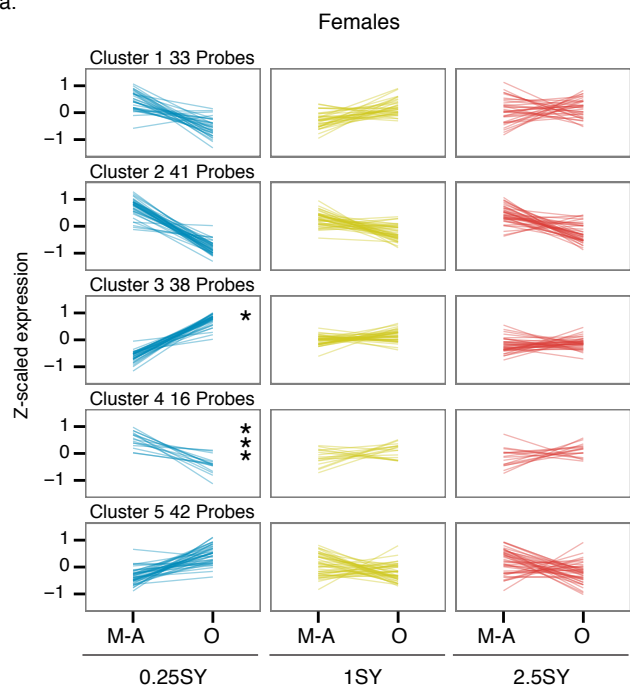

b.

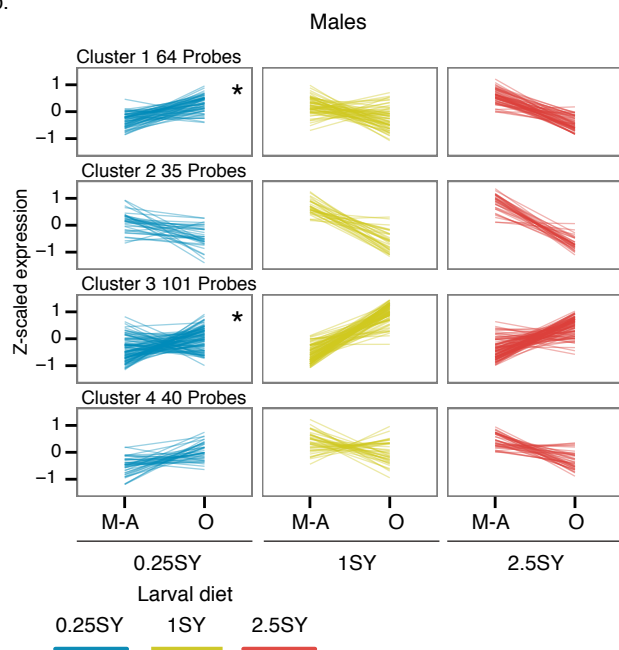

Supplement: Supplementary file 2 — Expression profiles of clusters of probes showing an interaction between larval diet and age in females (a) and males (b). Colour indicates larval diet (blue: 0.25SY, green: 1SY, red: 2.5SY) and the x-axis indicates larval diet and age (M-A: middle-aged and O: old). We identified five clusters in females (a) and four clusters in males (b), with no significant overlap between the male and female clusters, and with little significant GO annotation. These clusters revealed that the interaction between larval diet and age in both sexes was primarily due to 0.25SY-raised flies showing attenuated (males) or opposite (females) changes in expression with increasing age relative to 1SY and 2.5SY flies. In males, three of the clusters (C1,C2 & C4) were down-regulated with age in 1SY and 2.5SY-raised flies but up-regulated (C1 – proteasome complex, C4) or unchanging (C2– no annotation) in flies raised on the 0.25SY diet. The remaining cluster C3, was associated with the mitochondrial envelope and was down-regulated with age in 1SY and 2.5SY-raised flies, but relatively constant in 0.25SY-raised flies. In females, all clusters showed relatively little change in expression with age in 1SY and 2.5SY-raised flies, but down (C1,C2, C4) or up-regulation (C3,C5) in 0.25SY-raised flies. The only significant GO annotation was found for C3 (iron ion binding) and C4 (heme binding and terms relating to mitochondrial components and ATP synthesis). M-A: Middle-aged; O: Old age. Clusters of probes with similar expression profiles were identified using k-means clustering. * indicates number of significant GO terms associated with a cluster *:1 term, **: 2: to 9 terms,***: 10 or more terms. (PDF 841 kb) [file 12864_2017_3968_MOESM2_ESM.pdf]

a.

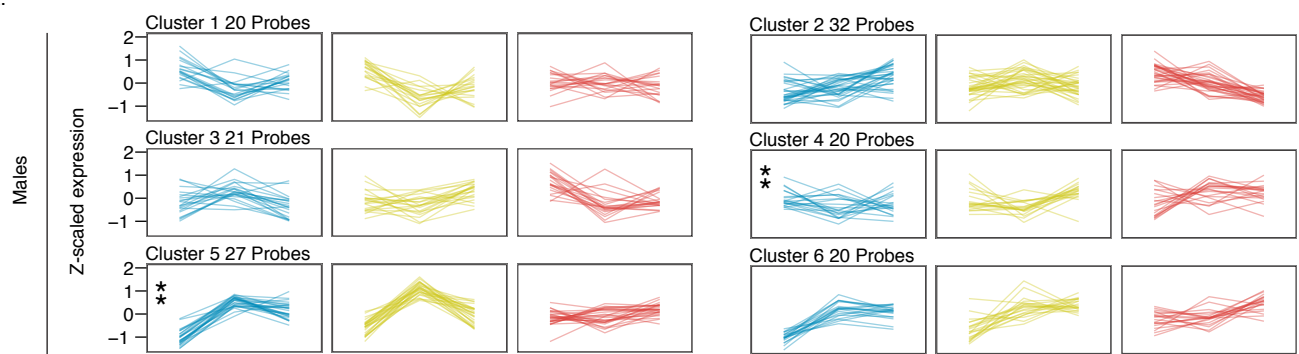

b.

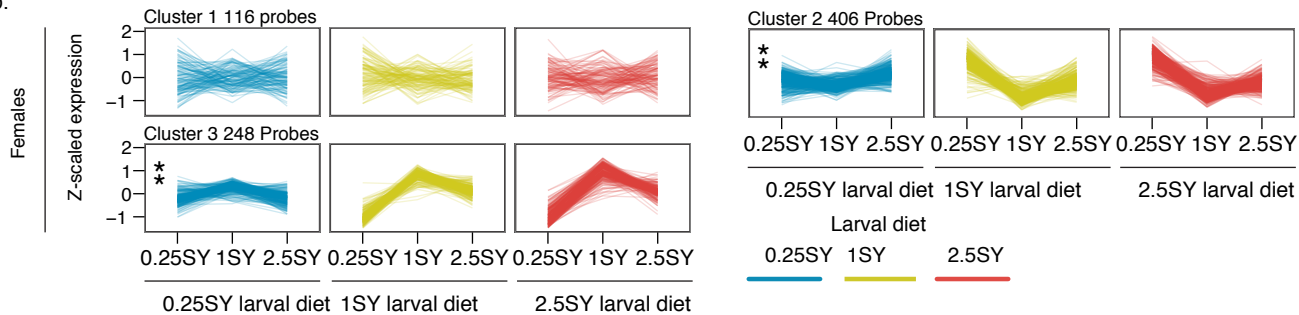

Supplement: Supplementary file 3 — Expression profiles of clusters of probes showing an interaction between larval diet and adult diet in males (a) and females (b). Colour indicates larval diet (blue: 0.25SY, green: 1SY, red: 2.5SY). In contrast to the genes affected by larval diet alone (L), there were many more probes showing L*A effects in females (770) than in males (140). In males, these probes broke down into six small clusters, only two of which (Clusters 4 and 5) had significant annotation. Cluster 4, which was up-regulated on the 1SY adult diet in 2.5SY-raised flies was annotated with responses to abiotic stimuli, particularly heat and oxygen, while cluster 5 which was down-regulated on the 1SY adult diet in 2.5SY-raised flies was associated with nucleobase metabolic processes. In females we identified three large clusters, two of which showed very distinct expression patterns and significant annotation. As with the “LT” clusters, these clusters were characterized by very similar expression patterns in 1SY and 2.5SY-raised flies, but distinct patterns in 0.25SY-raised flies. Cluster two was characterized by high expression on the 0.25SY adult diet for 1SY and 2.5SY-raised flies, but low expression in 0.25SY-raised flies. It was strongly associated with visual perception, circadian rhythm, regulation of behaviour, and metal ion transport. Cluster three was characterized by low expression on the 0.25SY adult diet for 1SY and 2.5SY-raised flies, but higher expression in 0.25SY-raised flies. This cluster was associated with nucleotide binding and female reproduction. Clusters of probes with similar expression profiles were identified using k-means clustering. * indicates number of significant GO terms associated with a cluster *:1 term, **: 2: to 9 terms,***: 10 or more terms. (PDF 2348 kb) [file 12864_2017_3968_MOESM3_ESM.pdf]

a.

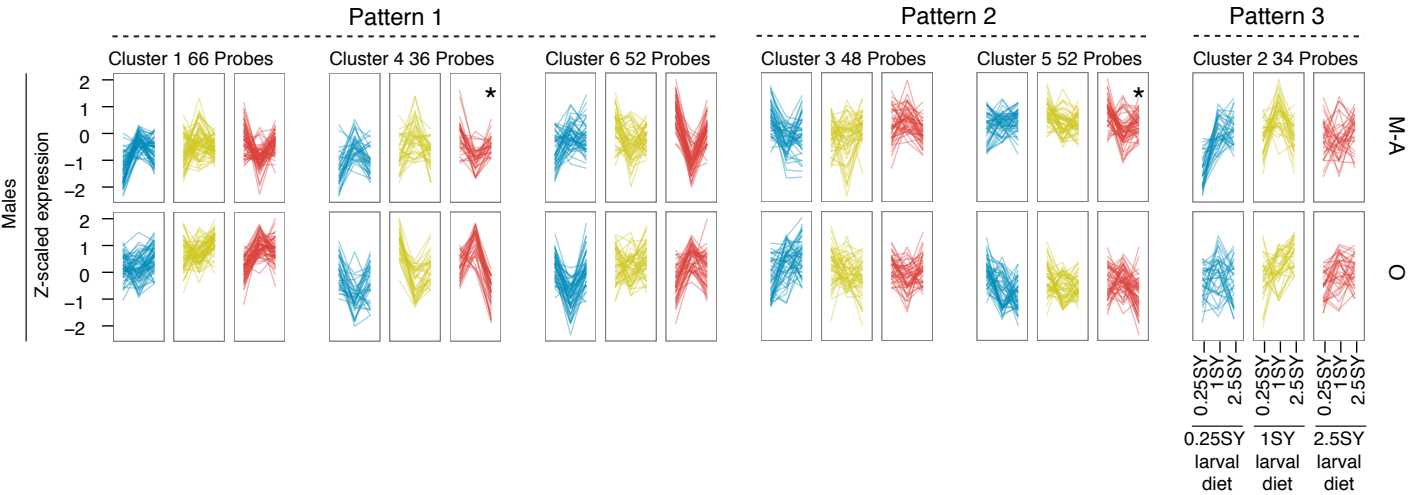

b.

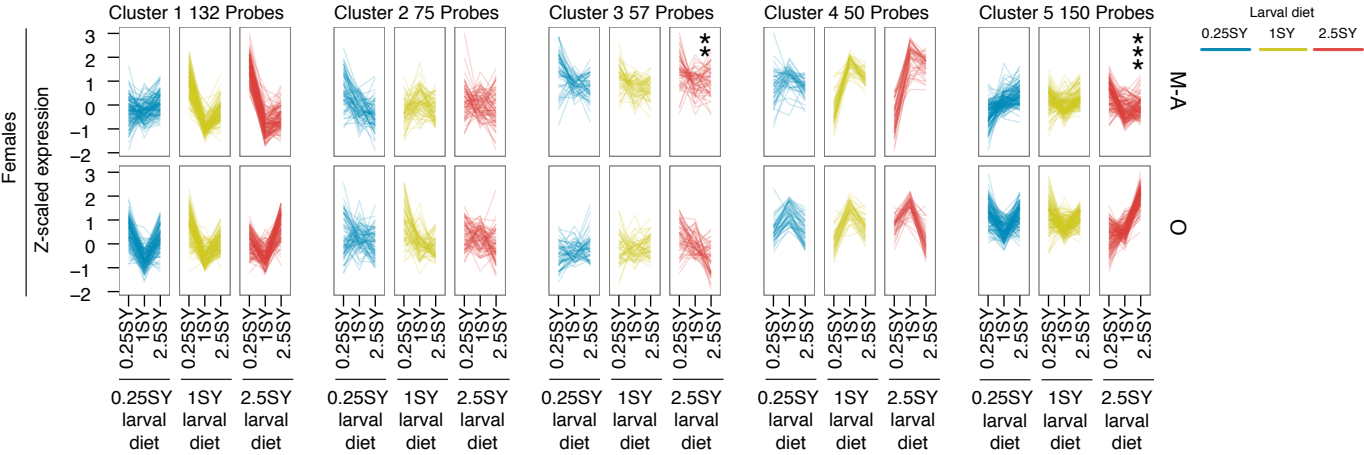

Supplement: Supplementary file 4 — Expression profiles of clusters of probes showing an interaction between larval diet (colour), adult diet (x-axis) and age in males (a) and females (b). Colour indicates larval diet (blue: 0.25SY, green: 1SY, red: 2.5SY), and the panels are split into middle age (M-A) and old-age (O). In males we identified six clusters of which only two possessed significant GO annotation – C4: endoplasmic reticulum and C5: post-mating behaviour. Clusters C1, C4 and C6 are characterized by distinct expression profiles for each larval diet across adult diets at middle-age, which are then inverted in old age, while clusters C3 & C5 are characterized by inversions in expression profiles across adult diets in age classes in 2.5SY-raised flies only. Finally, C2 shows distinct expression profiles in middle-age for 0..25SY & 1SY-raised flies, but no clear expression pattern in other ages or in 2.5SY-raised flies. In females, we identified five clusters each of which displayed distinct expression profiles and only two of which were annotated with GO terms (C3 and C5). As opposed to the males, we saw no evidence of inversion of responses to adult diet with increasing age. Cluster 3, which is associated with peptidase activity shows a distinct expression profile in old-aged flies raised on the 2.5SY larval diet, while cluster 5, which is associated with various terms relating to immune function and development, also shows a distinct expression profile in old-aged flies raised on the 2.5SY larval diet, as well as in middle-aged flies raised on the 0.25SY larval diet. Clusters of probes with similar expression profiles were identified using k-means clustering. * indicates number of significant GO terms associated with a cluster *:1 term, **: 2: to 9 terms,***: 10 or more terms. (PDF 4145 kb) [file 12864_2017_3968_MOESM4_ESM.pdf]
